# Improved Catalytic Performance of Au/α-Fe_2_O_3_-Like-Worm Catalyst for Low Temperature CO Oxidation

**DOI:** 10.3390/nano9081118

**Published:** 2019-08-03

**Authors:** Qiuwan Han, Dongyang Zhang, Jiuli Guo, Baolin Zhu, Weiping Huang, Shoumin Zhang

**Affiliations:** Key Laboratory of Advanced Energy Material Chemistry (MOE), TKL of Metal and Molecule Based Material Chemistry, Department of Chemistry, Nankai University, Tianjin 300071, China

**Keywords:** Au/α-Fe_2_O_3_, worm-like, CO oxidation

## Abstract

The gold catalysts supported on various morphologies of α-Fe_2_O_3_ in carbon monoxide (CO) oxidation reaction have been studied for many researchers. However, how to improve the catalytic activity and thermal stability for CO oxidation is still important. In this work, an unusual morphology of α-Fe_2_O_3_ was prepared by hydrothermal method and gold nanoparticles were supported using a deposition-precipitation method. Au/α-Fe_2_O_3_ catalyst exhibited great activity for CO oxidation. The crystal structure and microstructure images of α-Fe_2_O_3_ were carried out by X-ray diffraction (XRD) and scanning electron microscopy (SEM) and the size of gold nanoparticles was determined by transmission electron microscopy (TEM). X-ray photoelectron spectra (XPS) and Fourier transform infrared spectra (FTIR) results confirmed that the state of gold was metallic. The 1.86% Au/α-Fe_2_O_3_ catalyst calcined at 300 °C had the best catalytic performance for CO oxidation reaction and the mechanism for CO oxidation reaction was also discussed. It is highly likely that the small size of gold nanoparticle, oxygen vacancies and active sites played the decisive roles in CO oxidation reaction.

## 1. Introduction

Nowadays, environmental pollution has become a severe problem that cannot be ignored. Carbon monoxide (CO) is one of the common toxic gases in air pollution and its presence has a very bad influence on the environmental management and human health. Hence, it is definitely necessary to deal with the emission of CO. Usually, CO comes from automobile engines, fossil fuels, industrial chemical combustion and so on. The familiar effective controlling method is to convert CO into CO_2_ [1,2,3,4].

Gold nanocatalysts catalyzing CO oxidation reaction have attracted widespread concern among scientists since it was first reported by Haruta et al. [5] in the late 1980s. Many factors, including the size of gold particles, the property of support material and the prepared method and so on, have great influence on the catalytic activity [6]. Especially, the morphology of support has a non-negligible effect on the interaction between gold and support, further influencing the catalytic performance of gold catalysts. Jia et al. [7] investigated the effect of morphology of Ceria support on the activity of Au/CeO_2_ for CO oxidation. The results showed Au/nanopolyhedra-CeO_2_ had the better performance at low temperature, while Au/nanorod-CeO_2_ was the best catalyst at high temperature. Guczi et al. [8] interpreted that the activity of Au/oxide perimeter depended not only on the particle size but also on the morphology of the oxide component, likely amorphous structure. Li et al. [9] synthesized Au/CeO_2_-TiO_2_-nanorods and Au/CeO_2_-TiO_2_-nanoparticles by hydrothermal and co-precipitation methods, respectively. The catalytic results reported that Au/CeO_2_-TiO_2_-nanorods were more active than Au/CeO_2_-TiO_2_-nanoparticles because of the dominated surface structure of CeO_2_-TiO_2_ support.

A mass of papers have reported that the catalysts with gold supported on reducible oxide exhibit higher activities, such as Fe_2_O_3_, TiO_2_, Co_3_O_4_ and so on [10,11,12]. Hematite (α-Fe_2_O_3_), relying on large oxygen storage, narrow band gaps, low cost, earthly abundant and environmentally friendly properties, has often been chosen as support and loads gold to explore its catalytic activities for CO oxidation, photocatalysis, water-gas shift reaction and so on [2,13,14,15,16]. In these catalytic reactions, the structure or morphology of hematite plays an important role in catalytic performance [17,18,19], which is interesting for researchers and has been studied up to now. Zeng et al. [20] fabricated Au/α-Fe_2_O_3_-hollow catalyst by a hydrothermal-thermal decomposition process and experimental results declared that the Au/α-Fe_2_O_3_-hollow catalyst showed better catalytic performance for CO oxidation compared to other catalysts, where the morphologies of α-Fe_2_O_3_ were spindle, rod and hollow rod. After that, Zhang et al. [2] prepared the Ag/Fe_2_O_3_ catalyst derived from metal-organic framework (MOF), which had higher surface area and showed high catalytic activity for CO oxidation. At the same time, Shunsuke Tanaka et al. [21] reported another type of Fe_2_O_3_ catalyst supported gold for CO oxidation, in which Fe_2_O_3_ was prepared using an asymmetric PS-b-PAA-b-PEG triblock copolymer template. And the catalyst exhibited better catalytic activity compared to commercial Au/Fe_2_O_3_. Although these M/Fe_2_O_3_-modified (M = Au, Ag) catalysts show improved catalytic activity, it is still essential to make full conversion temperature lower in CO oxidation reaction by an easy prepared method and make catalysts own great catalytic performance in various reactions.

In this work, a novel worm-like α-Fe_2_O_3_ support was prepared via hydrothermal method and then was supported gold nanoparticles by a deposition-precipitation method to explore the catalytic activity for CO oxidation. The gold nanoparticles were distributed uniformly on the surface of α-Fe_2_O_3_. And experimental results showed that the Au/α-Fe_2_O_3_ catalyst possessed excellent catalytic activity for CO oxidation reaction. The gold nanoparticles in catalyst played an important role, which provided more active center and improved the catalytic activity for CO oxidation.

## 2. Materials and Methods

### 2.1. Materials

All chemical reagents were analytical grade and were used directly without any purification. Ferric chloride hexahydrate (FeCl_3_·6H_2_O, 99.0%) and glycol were purchased from Chemical Reagent Supply and Marketing Company, Tianjin, China. Ethanol was purchased from Guangfu Technology Development Co. Ltd., Tianjin, China. Urea was purchased from Wind Ship Chemical Reagent Technology Co. Ltd., Tianjin, China. Oleylamine and sodium hydroxide (NaOH, 96%) was purchased from the Aladdin Industry Corporation, Shanghai, China. Chloroauric acid (HAuCl_4_) was purchased from Masco Chemical Co. Ltd., Tianjin, China.

### 2.2. Preparation of α-Fe_2_O_3_ Support

The α-Fe_2_O_3_ support was prepared by a hydrothermal method. Typically, 2.4 g of FeCl_3_·6H_2_O was added into 60 mL of mixing solution of ethanol and glycol, whose volume ratio was 3:1. After FeCl_3_·6H_2_O was dissolved, 1.06 g of urea and 4 mL of oleylamine were added successively under constant stirring. The system was stirred for 1 h and the solution was then transferred into a 100 mL of Teflon-lined stainless steel autoclave. The autoclave was heated in an oil bath at 180 °C for 12 h. After the autoclave was cooled to room temperature naturally, the precipitate was washed with distilled water and ethanol several times, dried at 80 °C for 12 h and calcined at 600 °C for 150 min in air.

### 2.3. Preparation of Au/α-Fe_2_O_3_

Gold was supported onto the α-Fe_2_O_3_ support by a deposition-precipitation method. In this method, a certain amount of HAuCl_4_ solution was added dropwise to the α-Fe_2_O_3_ aqueous solution (0.4 g of α-Fe_2_O_3_ in 100 mL distilled water). After being violently stirred for 10 min, the pH value of the suspension was adjusted to about 8 by adding NaOH solution (1 mol/L) dropwise, then stirred and aged at room temperature for 12 h. After that, the suspension was heated in a thermostat water bath at 90 °C for 4 h. Finally, the precipitate was washed several times with deionized water to remove Cl^−^ and then dried at 80 °C for 12 h. The mass fraction of Au in sample was described as x% Au/α-Fe_2_O_3_ (x = 0.62, 1.86, 2.72, 3.59).

### 2.4. Characterization

The actual loadings of Au in samples were measured by inductively coupled plasma optical emission spectrometry (ICP-OES, Spectro, SpectroBlue, Germany). The X-ray diffraction (XRD) patterns were obtained using Rigaku D/max-2500 (Rigaku, Japan) X-ray diffractometer (Cu Κα, λ = 1.5418 Å) to identify the crystal phase of catalysts. Scanning electron microscopy (SEM) images of the samples were got using a JSM-7500F microscope (JEOL, Japan). Transmission electron microscopy (TEM) and high resolution (HR) TEM images were obtained using a Tecnai G2F20 microscopy (FEI, Hillsboro, OR, USA) operated at 200 kV. Ultraviolet-Visible diffuse reflectance spectra (UV-Vis DRS) were collected using a SHIMADZU UV-3600 spectrophotometer (Shimadzu, Japan). The Fourier transform infrared spectra (FTIR) were recorded using a Nicolet MAGNA-IR 560 spectrometer (Nicolet, Wisconsin, USA). X-ray photoelectron spectra (XPS) were accepted using a Kratos Axis Ultra DLD Spectrometer (Kratos Analytical Ltd., Manchester, UK) with a monochromator of Al Kα source to determine the chemical states of Au and Fe. The H_2_ temperature-programmed reduction profiles (H_2_-TPR) were tested on a ChemiSorb 2720 (Micromeritics, Georgia, GA, USA) and the temperature programmed desorption profiles of ammonia (NH_3_-TPD) were obtained using a chemBET TPD (Quantachrome, Florida, FL, USA).

### 2.5. CO Oxidation Catalytic Activity

The catalytic activity was evaluated using a fixed-bed flow millireactor at atmospheric pressure. The catalyst (0.2 g) was diluted with quartz sand (17.6 g) and then was loaded in a stainless steel tube, whose inner diameter is 8 mm. The feed gas containing of 10% CO balanced with air passed through the reactor at a total flow rate of 36.3 mL/min. The reaction temperature gradient was 5 °C/min and the testing temperature range was 25–200 °C. The effluent gases were analyzed using an on-line GC-508A gas chromatography equipped with H_2_ as carrier gas. The conversion rate of CO was calculated to evaluate the activity, whose equation [22] was shown as follows:

(1) $$\mathrm{CO}\;\mathrm{Conversion}=\frac{\left[{\mathrm{CO}}_{2}\right]}{\left[\mathrm{CO}\right]+\left[{\mathrm{CO}}_{2}\right]}\times 100\%,$$

## 3. Results and Discussion

### 3.1. ICP and XRD

The actual content of gold in sample was measured by ICP. Compared to the theoretical content of 1%, 2%, 3% and 4%, the actual content were 0.62%, 1.86%, 2.72% and 3.59%, respectively. Clearly, 60–90% of gold was successfully loaded on the α-Fe_2_O_3_ support for all catalysts.

**Figure 1:**
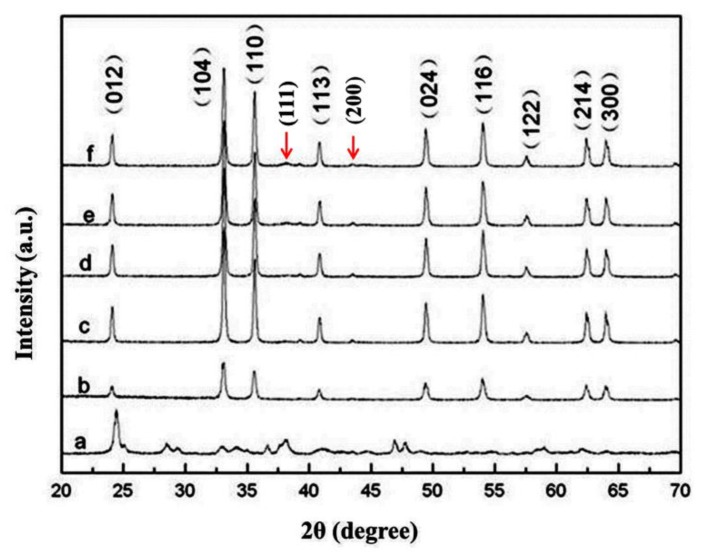
X-ray diffraction (XRD) patterns of (a) precursor, (b) α-Fe_2_O_3_ and various amount of Au/α-Fe_2_O_3_ catalysts ((c) 0.62%, (d) 1.86%, (e) 2.72%, (f) 3.59%) calcined at 300 °C for 2 h.

Figure 1 shows the XRD patterns of (a) precursor, (b) α-Fe_2_O_3_ and various amount of Au/α-Fe_2_O_3_ catalysts ((c) 0.62%, (d) 1.86%, (e) 2.72%, (f) 3.59%) calcined at 300 °C for 2 h, which revealed that the hexagonal α-Fe_2_O_3_ was prepared successfully after the calcination of precursor at 600 °C and the load of gold on the surface of α-Fe_2_O_3_ support did not change the phase of hematite. The precursor was made up of variety materials, whose diffraction peaks could be indexed to β-FeOOH, α-FeOOH, γ-Fe_2_O_3_ and α-Fe_2_O_3_ (JCPDS No. 34-1266, 29-0713, 39-1346 and 33-0664). The diffraction peaks of α-Fe_2_O_3_ perfectly corresponded to the hexagonal hematite (JCPDS No. 33-0664), of which the diffraction peaks at 2*θ* = 24.18°, 33.15°, 35.61°, 40.85°, 49.48°, 54.09°, 57.43°, 62.45° and 63.99° were indexed to {012}, {104}, {110}, {113}, {024}, {116}, {122}, {214} and {300} planes of hematite, respectively. The diffraction peaks of gold at 2*θ* = 38.2° and 44.5° were separately corresponded to {111} and {200} planes of Au fcc crystal (JCPDS No. 04-0784). In Figure 1 curves c–f, the peaks of gold were weak, which probably was caused from the small particle size or the relatively low gold content in catalysts [23]. Besides, it was clear that the peak intensity of hematite at 2*θ* = 33.15° and 35.61° in gold catalysts got stronger, which implied the crystalline of hematite increased after the addition of gold [22].

### 3.2. SEM and TEM

**Figure 2:**
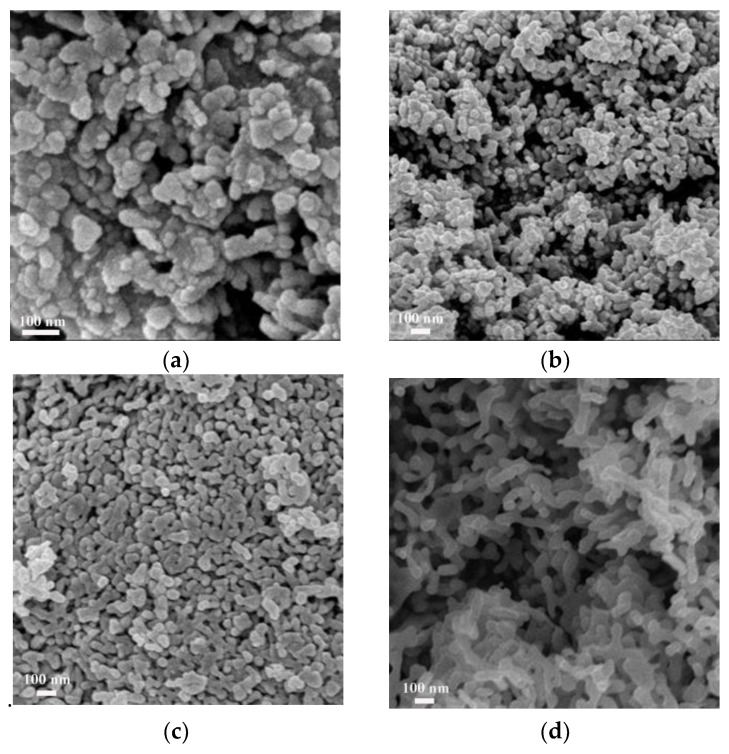
Scanning electron microscopy (SEM) images of α-Fe_2_O_3_ reacting for (**a**) 4 h, (**b**) 6 h, (**c**) 10 h and (**d**) 12 h at 180 °C.

**Figure 3:**
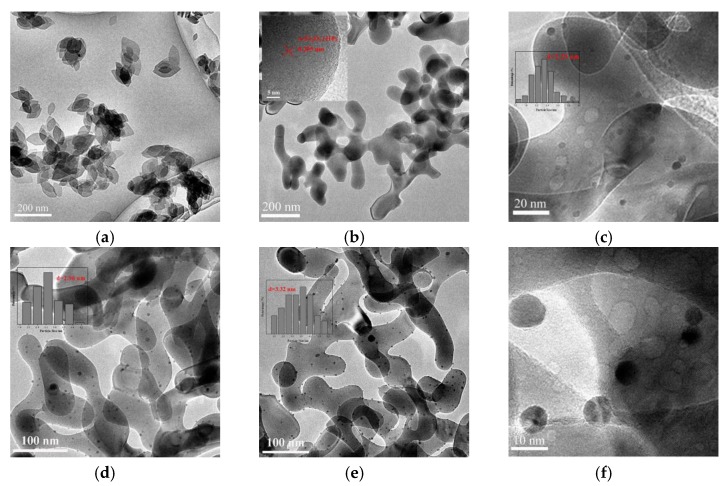
Transmission electron microscopy (TEM) images of (**a**) precursor; (**b**) α-Fe_2_O_3_; (**c**) 1.86%, (**d**) 2.72% and (**e**) 3.59% Au/α-Fe_2_O_3_ catalysts calcined at 300 °C; (**f**) 1.86% Au/α-Fe_2_O_3_ catalysts calcined at 500 °C.

The SEM and TEM images are shown in Figure 2 and Figure 3. As shown in Figure 2, the hydrothermal time played an important role in the morphology of hematite. Firstly, when it was heated at 180 °C for 4 h in oil bath, α-Fe_2_O_3_ was mainly made up of irregular particles. With the further increase of time, it could be seen that the morphologies of α-Fe_2_O_3_ support were increasingly like worm. While the hydrothermal time increased to 12 h, the worm-like α-Fe_2_O_3_ finally formed and it was used for all subsequent studies. Usually, the morphology of α-Fe_2_O_3_ obtained by hydrothermal method is spindle-shaped or spherical [24]. However, in this work, a novel worm-like α-Fe_2_O_3_ support was prepared by reacting for 12 h using hydrothermal method, whose specific surface area was 23.2 m^2^/g measured by BET. Its TEM image is shown in Figure 3b, of which the inset is the HR-TEM image of α-Fe_2_O_3_ support. It could be clearly seen that the lattice fringe was 0.255 nm, which was corresponded to the (110) plane of α-Fe_2_O_3_ [21,25]. The rest of TEM images in Figure 3 separately represents (a) precursor, (c) 1.86%, (d) 2.72% and (e) 3.59% Au/α-Fe_2_O_3_ catalysts calcined at 300 °C; (f) 1.86% Au/α-Fe_2_O_3_ catalysts calcined at 500 °C. The morphology of precursor in Figure 3a was like-leaf before changing into hematite. In Figure 3c–e, gold nanoparticles were distributed on the surface of hematite support in various gold catalysts. The insets of Figure 3c–e shows the corresponding size distributions of gold nanoparticles, which indicated the average diameters of gold nanoparticles were 2.29, 2.96 and 3.32 nm in 1.86%, 2.72% and 3.59% Au/α-Fe_2_O_3_ catalysts, respectively. Additionally, some agglomerate gold nanoparticles occasionally appeared on the surface of 2.72% Au/α-Fe_2_O_3_ and 3.59% Au/α-Fe_2_O_3_ catalysts, which was probably due to the increase content of gold in catalysts compared to that in 1.86% Au/α-Fe_2_O_3_ catalyst. It also could be seen that the size of gold in Figure 3f increased to around 7 nm due to the calcination. The size of gold nanoparticles has a great effect on the catalytic activity. As we know, the smaller gold particle size improves the catalytic performance in CO oxidation reaction.

### 3.3. UV-Vis DRS and FTIR

**Figure 4:**
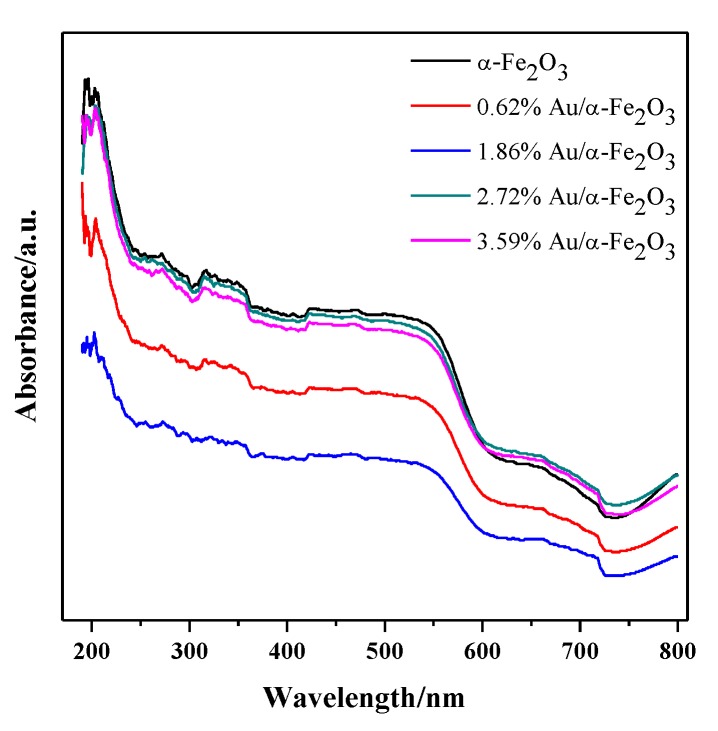
Ultraviolet-Visible diffuse reflectance spectra (UV-Vis DRS) of α-Fe_2_O_3_ and Au/α-Fe_2_O_3_ catalyst calcined at 300 °C.

The UV-Vis spectra of α-Fe_2_O_3_ and Au/α-Fe_2_O_3_ catalysts with various gold loadings calcined at 300 °C for 2 h are shown in Figure 4. The absorption of α-Fe_2_O_3_ at wavelengths (200–800 nm) were usually attributed to three kinds of electronic transitions [26]. The absorption peaks at 200–400 nm mainly resulted from the charge transfer from ligand to metal and partly from the contribution of Fe^3+^ ligand field transitions. The obvious peak at 530–570 nm was considered to form mainly by the pair excitation process of α-Fe_2_O_3_. And the peaks at 600–900 nm could be corresponded to the d–d transition [26,27]. The peak of gold loaded on support normally occurs at 500–570 nm due to the localized surface plasmon resonance of Au nanoparticles [4]. There was no obvious peak of gold, which might be overlapped with the peak of α-Fe_2_O_3_ at 530–570 nm. This weak gold peak further indicated the small gold nanoparticle size in catalysts, which was consistent with TEM results (Figure 3). Besides, it was apparent that the peaks of Au/α-Fe_2_O_3_ catalyst all had an obvious red-shift compared to that of α-Fe_2_O_3_ support and the 1.86% Au/α-Fe_2_O_3_ catalyst had the largest red-shift among these gold catalysts. The red-shift action was caused probably from the reduction of electron density in the gold nanoparticles owing to the chemical interactions with neighboring hematite, in which the electrons transformed from the cluster to the surrounding matrix [28].

**Figure 5:**
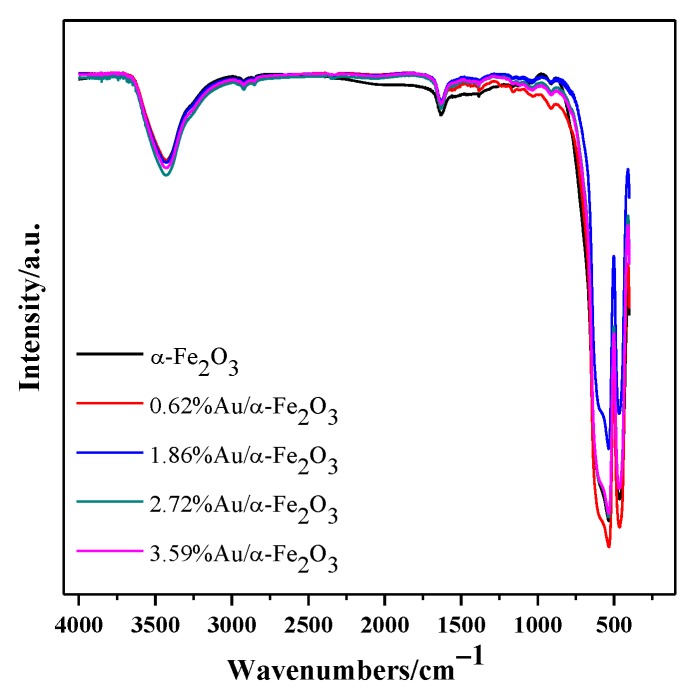
The Fourier transform infrared (FTIR) spectra of α-Fe_2_O_3_ and Au/α-Fe_2_O_3_ catalyst calcined at 300 °C.

Figure 5 shows the FTIR spectra of α-Fe_2_O_3_ and Au/α-Fe_2_O_3_ calcined at 300 °C for 2 h. The obviously broad peak at about 3420 cm^−1^ was attributed to the O–H stretching vibration of absorbed water and the shoulder peak at 1630 cm^−1^ was ascribed to the H–OH bending vibration of absorbed water [29,30]. The two sharp peaks at 460 cm^−1^ and 530 cm^−1^ were mainly due to the Fe–O vibration of α-Fe_2_O_3_ [31,32]. The FTIR spectra of gold catalysts were similar with that of support, indicating the addition of gold has a little effect on the bond of hematite.

### 3.4. XPS

**Figure 6:**
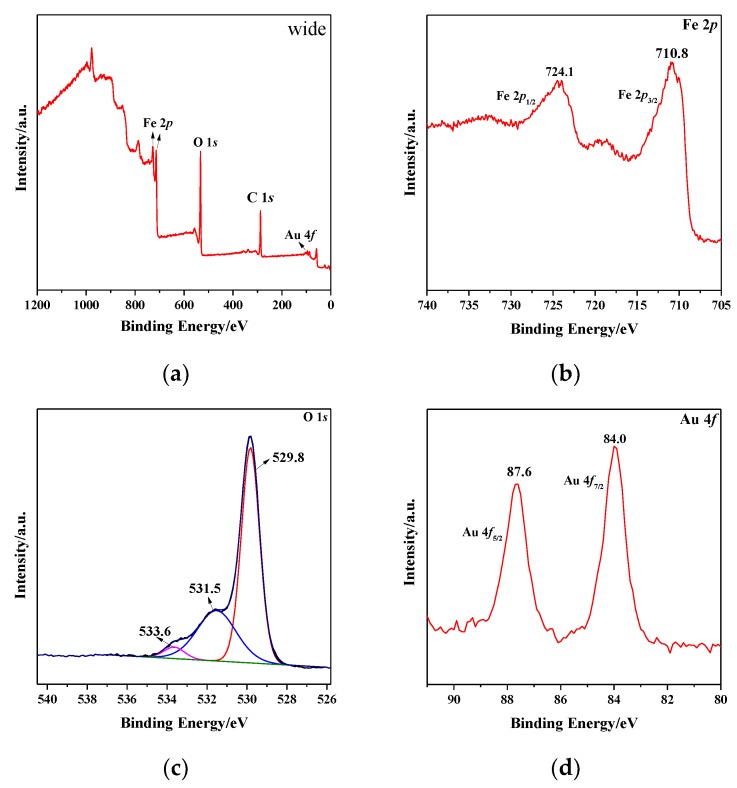
(**a**) The wide pattern, (**b**) Fe 2*p*, (**c**) O 1*s*, (**d**) Au 4*f* XPS patterns of 1.86% Au/α-Fe_2_O_3_ catalyst calcined at 300 °C.

The oxidation states and the surface elemental composition of catalysts were further confirmed by XPS and the XPS results of 1.86% Au/α-Fe_2_O_3_ calcined at 300 °C for 2 h were shown in Figure 6. Figure 6a shows the full spectrum of Au/α-Fe_2_O_3_, where the peaks of Fe, O, C and Au were assigned, respectively. Especially, the element C came from the hydrocarbon compound in instrument and the reason for the small hump of gold was probably related to the low loading. For the Fe 2*p* XPS spectrum in Figure 6b, the peaks at 710.8 eV and 724.1 eV corresponded to Fe 2*p*_3/2_ and Fe 2*p*_1/2_, respectively. The binding energy (BE) value for Fe 2*p*_3/2_ peak was consistent with the value for ferric oxides reported by A.P. Grosvenor et al. [33] and the Fe^3+^ cation occupied octahedral sites. Figure 6c shows the high resolution XPS spectrum of O 1*s* in Au/α-Fe_2_O_3_. The main peak at 529.8 eV corresponded to the lattice oxygen of hematite and the other two peaks at 531.5 eV and 533.6 eV were attributed to the oxygen vacancies in the structure and the surface OH^−1^ groups [34,35], respectively. The state of gold in Au/α-Fe_2_O_3_ catalyst was further determined by the Au 4*f* XPS spectra in Figure 6d. It could be seen that the BE of Au 4*f*_7/2_ was 84.0 eV and the BE of Au 4*f*_5/2_ was 87.6 eV, which corresponded to the metallic gold [36]. The result was consistent with the FTIR analytical result. Besides, Overbury et al. [37] has reported that the valence of gold had an impact on the catalytic property of catalyst and the metallic gold played an improved role in catalyzing CO oxidation.

### 3.5. H_2_-TPR and NH_3_-TPD

**Figure 7:**
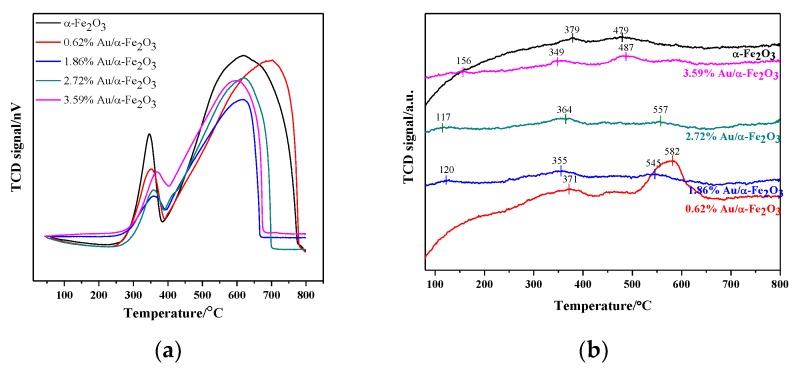
(**a**) H_2_ temperature-programmed reduction (H_2_-TPR) profiles and (**b**) temperature programmed desorption of ammonia (NH_3_-TPD) profiles of α-Fe_2_O_3_ and Au/α-Fe_2_O_3_ catalyst calcined at 300 °C.

The H_2_-TPR results evaluated the reduction behaviors of α-Fe_2_O_3_ support and Au/α-Fe_2_O_3_ catalyst calcined at 300 °C for 2 h, which were shown in Figure 7a. There were two reduction peaks in the TPR curve of hematite, which were separately located at about 340 and 610 °C. The reduction peak around 340 °C was assigned to the reduction of Fe_2_O_3_ to Fe_3_O_4_, while another stronger peak around 610 °C could be attributed to the reduction of Fe_3_O_4_ to FeO and Fe^0^ [38,39]. In the reductive process of Fe_2_O_3_ to Fe_3_O_4_, the reductive temperature (340 °C) in this work compared to that (380 °C) reported by Zhang et al. [40] decreased, which declared that the hematite presented stronger reducibility.

Au/α-Fe_2_O_3_ catalysts with various gold loadings had two reduction peaks and their reduction processes were identical with α-Fe_2_O_3_. However, the first peak position in Au/α-Fe_2_O_3_ shifted to higher temperature compared with pure α-Fe_2_O_3_ and the second peak position was also different from α-Fe_2_O_3_. In these latter peaks, the 1.86% Au/α-Fe_2_O_3_ catalyst had the lowest temperature in achieving complete reduction, indicating that it had the most improved reducibility, which was beneficial to catalyze CO oxidation reaction.

The NH_3_ desorption curves of α-Fe_2_O_3_ and Au/α-Fe_2_O_3_ catalyst calcined at 300 °C were shown in Figure 7b. It was clear that there were mainly three desorption peaks in the temperature range of 100–800 °C for these catalysts. The peaks in all catalysts around 110–200 °C were attributed to the weak acid sites and physiosorbed NH_3_ and the peaks around 300–400 °C were ascribed to the medium acid sites. While in the high-temperature area, the peaks around 450–650 °C were elaborated to the strong acid sites [41,42]. Considering the desorption peaks at low temperature, the 1.86% and 2.72% Au/α-Fe_2_O_3_ had a relatively weaker acidity among these catalysts. And in the peaks of all catalyst at high temperature, the 0.62% Au/α-Fe_2_O_3_ catalyst presented stronger acidity on account of the higher desorption temperature. The acid-base property is related to the oxidizing ability and the strong acidity of catalyst has the disadvantageous effect on the formation of active oxygen. Hence, the catalyst with weaker acidity would be conducive to the CO oxidation reaction.

### 3.6. CO Oxidation Catalytic Activity

**Figure 8:**
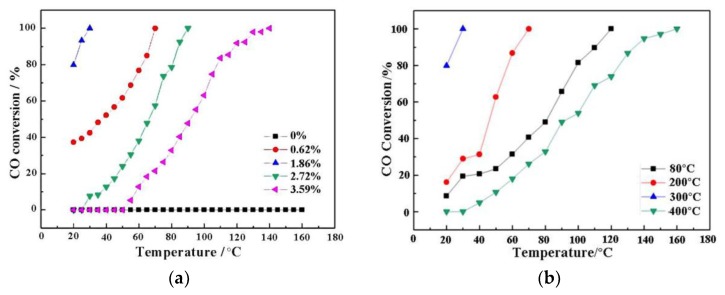
(**a**) Catalytic activities of different amount of Au/α-Fe_2_O_3_ catalysts; (**b**) Catalytic activities of 1.86% Au/α-Fe_2_O_3_ calcined at different temperature.

The relation schema between CO conversion rate and reaction temperature of Au/α-Fe_2_O_3_ catalyst was shown in Figure 8a. As seen in it, the α-Fe_2_O_3_ support had no catalytic activity for CO oxidation, while 1.86% Au/α-Fe_2_O_3_ had the best catalytic ability with the T_100_ (T_100_, the temperature that CO conversion rate is 100%) of 30 °C. And the T_100_ of 0.62%, 2.72% and 3.59% Au/α-Fe_2_O_3_ were 70, 90 and 140 °C, respectively. According to these catalytic results, it could be easily deduced that the addition of gold had a great influence on the catalytic activity. Moreover, when the content of gold was higher than 1.86, the catalytic activity began to decrease, which might be related to the decrease of active center. As shown in the characterization results of UV-Vis and NH_3_-TPD, 1.86% Au/α-Fe_2_O_3_ catalyst equipped with stronger interaction between gold and support, which could promote the formation of more active center. And the weaker acidity of 1.86% Au/α-Fe_2_O_3_ would accelerate the generation of active oxygen, which was benefit for the CO oxidation. The catalytic performance of Au/α-Fe_2_O_3_-like-worm in this work was also compared with other gold catalysts supported on Fe_2_O_3_ and CeO_2_ reported in the literature for CO oxidation, which was shown in Table 1. From Table 1, it could be seen that the Au/α-Fe_2_O_3_-like-worm catalyst performed the relatively high catalytic activity among these catalysts for CO oxidation. And the specific rate of Au/α-Fe_2_O_3_-like-worm (2.61 mol_CO_g_Au_^−1^h^−1^) was 26.1 and 2.2 times higher than those of Au/Fe_2_O_3_-WGC (0.10 mol_CO_g_Au_^−1^h^−1^) and Au/commercial Fe_2_O_3_ (1.21 mol_CO_g_Au_^−1^h^−1^) catalysts. It is well known that several factors, such as gold particle size, preparation method and the choice of support may give rise to the different performance of gold catalysts [43].

Calcination is a good measure to prepare high-performance catalyst and different calcined temperature has diverse influence on catalyzing CO oxidation. Figure 8b was the CO conversion curves of 1.86% Au/α-Fe_2_O_3_ catalyst calcined at different temperature. When the reaction temperature was 20 °C, the conversion rate of catalyst calcined at 80, 200, 300 and 400 °C were 10%, 20%, 80% and 0%, respectively. And the T_100_ of these catalysts were separately 120, 70, 30 and 160 °C. Clearly, the catalyst calcined at 300 °C presented the best catalytic activity. Boccuzzi et al. [46] has reported that the impact of calcined temperature on catalytic activity is mainly ascribed to the size of gold particles. The smaller size of gold nanoparticles could provide more effective defects, such as edge, corner, step and so on. Hence, the decreased catalytic activity of catalyst calcined at 400 °C was possibly related to the sinter of gold nanoparticles.

**Figure 9:**
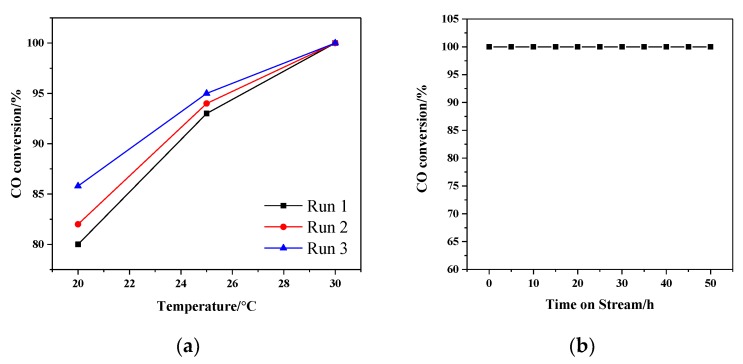
(**a**) Reproducibility of 1.86% Au/α-Fe_2_O_3_ catalyst calcined at 300 °C for 2 h in CO oxidation reaction; (**b**) the lifetime test of 1.86% Au/α-Fe_2_O_3_ catalyst calcined at 300 °C for 2 h at 30 °C (T_100_).

**Figure 10:**
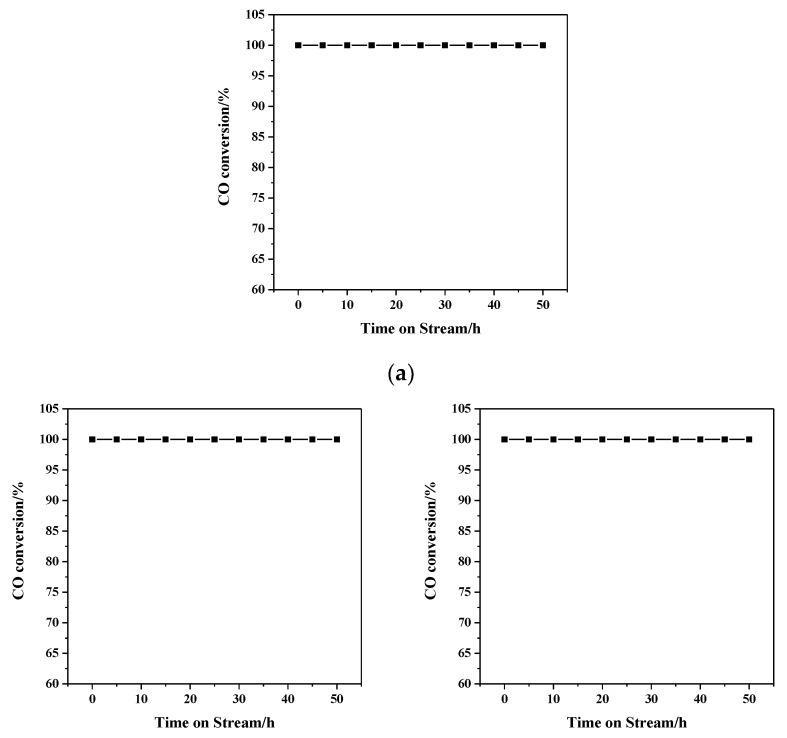
The high-temperature stability of 1.86% Au/α-Fe_2_O_3_ catalyst calcined at 300 °C for 2 h, at the CO oxidation reaction temperature of (**a**) 100 °C, (**b**) 140 °C, (**c**) 180 °C.

The repeatability, persistence and thermostability of samples were further studied. The repeated test curves of 1.86% Au/α-Fe_2_O_3_ catalyst calcined at 300 °C were shown in Figure 9a. After finishing the first circulation, the test was restarted from room temperature. In the third repeated test, the CO conversion rate still stayed at 100% at the reaction temperature of 30 °C. Therefore, the catalyst had a high repeatability in catalyzing CO oxidation. The persistent test of catalyst was reported in Figure 9b and the reaction was kept for 60 h at 30 °C (T_100_). This result showed that there was no decline in catalytic activity, which strongly proved the catalyst had a long service life. The high-temperature stability tests were shown in Figure 10. 1.86% Au/α-Fe_2_O_3_ catalyst calcined at 300 °C was run for 50 h at 100, 140 and 180 °C, respectively. Excitedly, the conversion rates of all catalysts were maintained at 100% after keeping reaction for 50 h, indicating that the Au/α-Fe_2_O_3_ catalyst with high temperature-stable was definitely prospective in industrial application for CO oxidation.

**Figure 11:**
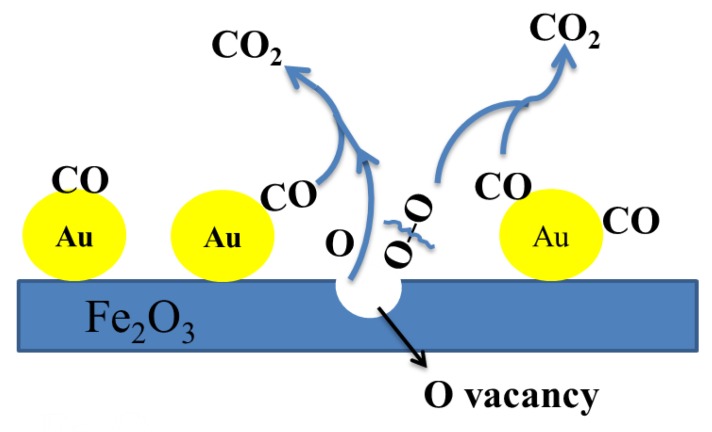
Schematic diagram of the possible CO oxidation mechanism for Au/α-Fe_2_O_3_.

The CO oxidation reaction mechanisms for metal oxide supported gold were highly debated in the literatures [47,48,49]. In this work, the possible reaction mechanism of hematite supported gold catalyst was showed in Figure 11 and described as follows. Further research would be carried out to investigate the detailed reaction mechanism. CO molecule was absorbed on the surface of gold in the Au/α-Fe_2_O_3_ catalyst and the catalyst was activated. The lattice oxygen in α-Fe_2_O_3_ connected with CO absorbed on the surface of gold, leaving oxygen defects in hematite. Then the defects were occupied by O_2_ in air and the O_2_ molecule sequentially reacted with the CO absorbed on gold to form CO_3_ structure. Followingly, the CO_3_ molecule decomposed to CO_2_ and the O vacancy in hematite was filled with the O of gas-phase O_2_ [50,51,52]. Hence, more defects in hematite might more largely improve the conversion of CO to CO_2_ and promote the catalytic ability of catalyst.

## 4. Conclusions

In summary, a novel Au/α-Fe_2_O_3_-like-worm catalyst has been prepared firstly by hydrothermal and deposition-precipitation method. It has been shown that the 1.86% Au/α-Fe_2_O_3_ catalyst calcined at 300 °C had the best catalytic activity in CO oxidation due to the small size of Au particle and more active center. The catalyst achieved complete CO conversion at a temperature of 30 °C. And the conversion rate of 1.86% Au/α-Fe_2_O_3_ was still 100% after running 60 h at 30 °C, indicating the high stability of Au/α-Fe_2_O_3_. The Au/α-Fe_2_O_3_ catalyst owned effective activity for low-temperature CO oxidation and excellent stability, which suggested that it is prospective to be used to control the emission of CO in industrial application. Moreover, the study of novel worm-like α-Fe_2_O_3_ would shed new light on wide application of α-Fe_2_O_3_ in other catalytic reactions. The excellent performance of Au/α-Fe_2_O_3_-like-worm is expected to spur further development for gold catalysts for low-temperature CO oxidation.

## Figures and Tables

**Table 1:** Comparison of catalytic performance of different gold catalysts for CO oxidation.

Catalyst	Au Loading (wt.%)	Au Particle size (nm)	Specific Rate (mol_CO_g_Au_^−1^h^−1^)	T_100_ (K)	Reference
Au/α-Fe_2_O_3_-like-worm	1.86	2.3	2.61	303	This work
Au/Fe_2_O_3_-WGC	0.5	3.7	0.10	623	[43]
Au/Fe_2_O_3_	1.0	7.4	0.94	423	[43]
Au/commercial Fe_2_O_3_ (Fluka)	0.5	1–5	1.21	--	[44]
Au/Fe_2_O_3_-nanorod	0.5	1–5	3.99	--	[44]
Au/Fe_2_O_3_-mesoporous	7.9	3–10	0.30	523	[21]
Au/CeO_2_	3.2	5.4	0.26	320	[45]
